# Regularized Latent Class Analysis for Polytomous Item Responses: An Application to SPM-LS Data

**DOI:** 10.3390/jintelligence8030030

**Published:** 2020-08-14

**Authors:** Alexander Robitzsch

**Affiliations:** 1 IPN—Leibniz Institute for Science and Mathematics Education, D-24098 Kiel, Germany; robitzsch@leibniz-ipn.de; 2 Centre for International Student Assessment (ZIB), D-24098 Kiel, Germany

**Keywords:** regularized latent class analysis, regularization, fused regularization, fused grouped regularization, distractor analysis

## Abstract

The last series of Raven’s standard progressive matrices (SPM-LS) test was studied with respect to its psychometric properties in a series of recent papers. In this paper, the SPM-LS dataset is analyzed with regularized latent class models (RLCMs). For dichotomous item response data, an alternative estimation approach based on fused regularization for RLCMs is proposed. For polytomous item responses, different alternative fused regularization penalties are presented. The usefulness of the proposed methods is demonstrated in a simulated data illustration and for the SPM-LS dataset. For the SPM-LS dataset, it turned out the regularized latent class model resulted in five partially ordered latent classes. In total, three out of five latent classes are ordered for all items. For the remaining two classes, violations for two and three items were found, respectively, which can be interpreted as a kind of latent differential item functioning.

## 1. Introduction

There has been recent interest in assessing the usefulness of short versions of the Raven’s Progressive Matrices. Myszkowski and Storme (2018) composed the last 12 matrices of the Standard Progressive Matrices (SPM-LS) and argued that it could be regarded as a valid indicator of general intelligence *g*. As part of this special issue, the SPM-LS dataset that was analyzed in Myszkowski and Storme (2018) was reanalyzed in a series of papers applying a wide range of psychometric approaches.

Previous reanalyses of the SPM-LS dataset have in common that quantitative latent variable models were utilized. In this paper, discrete latent variable models (i.e., latent class models) are applied for analyzing the SPM-LS dataset. With discrete latent variable models, the analysis of types instead of traits is the primary focus (see von Davier et al. (2012) and Borsboom et al. (2016)). A disadvantage of discrete latent variable models is that they often have a large number of parameters to estimate. For example, latent class models result in item response probabilities that are allowed to vary across classes. Even with only a few classes, the number of estimated parameters is typically larger than parametric models with quantitative latent variables. Hence, model selection based on principles often favors quantitative latent variable models over discrete latent variable models. So-called regularization approaches automatically reduce the number of parameters to estimate (see Huang et al. (2017) or Jacobucci et al. (2016)) for the use of regularization in structural equation modeling and Tutz and Schauberger (2015) or Battauz (2019) in item response modeling). In this paper, these regularization approaches are applied in discrete latent variable models, and some extensions for polytomous data are proposed.

The paper is structured as follows. In Section 2, we give a brief overview of latent class analysis. In Section 3, regularized latent class analysis for dichotomous and polytomous data is introduced. In Section 4, we apply proposed models of Section 3 in a simulated data illustration. In Section 5, we apply regularized latent class analysis to the SPM-LS dataset. Finally, in Section 6, we conclude with a discussion.

## 2. Latent Class Analysis

Latent variable models represent discrete items by a number of latent variables (see Agresti and Kateri 2014 for an overview). These latent variables can be categorical or quantitative or a mixture of both. Quantitative latent variables are considered in factor analysis, structural equation models, or item response models. In this article, we focus on categorical latent variables. In this case, latent variables are labeled as latent classes and are extensively studied in the literature of latent class analysis (LCA; Collins and Lanza 2009; Langeheine and Rost 1988; Lazarsfeld and Henry 1968).

A latent class model (LCM) represents the multivariate distribution of *I* categorical items $X=(X_{1},\ldots ,X_{I})$ by a fixed number of *C* latent classes. Let *U* denote the latent class variable that takes one of the values 1,2,…,C. It is assumed that items $X_{i}$ are conditionally independent on the latent class variable *U*. This means that it holds that

(1) $$P\left(X=x|U=c\right)=\prod_{i=1}^{I}P\left(X_{i}=x_{i}|U=c\right)\;\mathrm{for}\;\;x=\left(x_{1},\ldots ,x_{I}\right).$$

The multivariate probability distribution is then given as a mixture distribution

(2) $$P\left(X=x\right)=\sum_{c=1}^{K}P\left(U=c\right)\prod_{i=1}^{I}P\left(X_{i}=x_{i}|U=c\right).$$

Applications of LCMs to intelligence tests can be found in Formann (1982) or Janssen and Geiser (2010).

### 2.1. Exploratory Latent Class Analysis for Dichotomous Item Responses

In this subsection, we describe the LCM for dichotomous items. Let $p_{ic}=P\left(X_{i}=1|U=c\right)$ denote the item response probability for correctly solving item *i* if a person is located in class *c*. In the estimation, these bounded parameters ($p_{ic}\in \left[0,1\right]$) are transformed onto the real line by using the logistic transformation (see also Formann 1982)

(3) $$P\left(X_{i}=x|U=c\right)=\frac{\exp(x\gamma_{ic})}{1+\exp(\gamma_{ic})}\;\left(x=0,1\right).$$

Note that $p_{ic}$ is a one-to-one function of $\gamma_{ic}$. For estimation purposes, it is sometimes more convenient to estimate models with unbounded parameters instead of estimating models with bounded parameters. For *I* items and *C* classes, I·C item parameters have to be estimated in the case of dichotomous items. In comparison to item response models (1PL model: one parameter, 2PL: two parameters, etc.), this results in many more parameters to be estimated. However, LCMs do not pose the assumption that classes are ordered, and no monotonicity assumptions of item response functions are posed.

Moreover, let $p_{c}=P\left(U=c\right)$ denote the probability that a person is in class *c*. As for item parameters, a logistic transformation is used to represent the class probabilities $p_{c}$ by parameters $\delta_{c}$. More formally, we set

(4) $$p_{c}=\frac{\exp(\delta_{c})}{1+\sum_{j=2}^{C}\exp\left(\delta_{j}\right)}\;\left(c=1,\ldots ,C\right),$$

where $\delta_{1}=0$. Because the probabilities sum to one, only C−1 distribution parameters have to be estimated. In total, the saturated distribution of *I* dichotomous items has $2^{I}-1$ free possible parameters, which is represented by I·C+C−1 parameters in the LCM with *C* classes.

LCMs can be interpreted as pure exploratory models because no structure of item response probabilities among classes is posed. Confirmatory LCMs assume additional equality constraints on item response probabilities (Finch and Bronk 2011; Nussbeck and Eid 2015; Oberski et al. 2015; Schmiege et al. 2018). Like in confirmatory factor analysis, it could be assumed that some items load only on some classes, which translates into equal item response probabilities. Cognitive diagnostic models can be seen as particular confirmatory LCMs (von Davier and Lee 2019).

It should be emphasized that restricted LCMs form the basis of almost all popular latent variable models for discrete item responses that are nowadays very popular. Formann (1982) suggested to represent the vector $\gamma =(\gamma_{11},\ldots ,\gamma_{1C},\ldots ,\gamma_{I1},\ldots \gamma_{IC})$ of item parameters as linear combinations $\gamma_{ic}=q_{ic}\alpha$(i=1,…,I;c=1,…,C) using a parameter vector α and known weight vectors $q_{ic}$. In addition, the distribution parameter $\delta =(\delta_{1},\ldots ,\delta_{C})$ is represented by $\delta_{c}=w_{c}\beta$ using a parameter vector β and known weight vectors $w_{c}$. The resulting so-called structured LCM (Formann and Kohlmann 1998) includes unidimensional and multidimensional 1PL and 2PL models as special cases as well as mixture item response models (Formann 2007). To accomplish this, continuous latent variables are approximated by a finite number of discrete latent classes. For example, a normally distributed latent variable is approximated by discrete latent classes (e.g., C=21 classes) whose probabilities are represented by only two components in α (i.e., the mean and the standard deviation as the first two moments). The usage of discrete latent classes can be interpreted as performing numerical integration with a fixed integration grid and applying the rectangle rule. Similar generalizations of restricted LCM were proposed by researcher von Davier (2008, 2010). In the rest of this article, we will focus on the simple exploratory LCM, although the proposed extension also applies to the more general structured latent class model.

In many applications, the allocation of persons to classes should be predicted by person variables Z (Collins and Lanza 2009). In more detail, class probabilities $p_{c}=P\left(U=c\right)$ are replaced by subject-specific conditional probabilities P(U=c|Z=z) (so-called latent class regression). These models further ease the interpretation of latent classes.

### 2.2. Exploratory Latent Class Analysis for Polytomous Item Responses

Now assume that there are *I* polytomous items and each item has $K_{i}+1$ nominal categories $0,1,\ldots ,K_{i}$. Item response probabilities are then given as $p_{ikc}=P\left(X_{i}=k|U=c\right)$ and are again transformed into unbounded parameters $\gamma_{ikc}$ by a logistic transformation. In more detail, it is assumed that

(5) $$P\left(X_{i}=x|U=c\right)=\frac{\exp(\gamma_{ixc})}{1+\sum_{k=1}^{K_{i}}\exp\left(\gamma_{ikc}\right)}\;\left(x=0,1,\ldots ,K_{i}\right),$$

where $\gamma_{i0c}=0$ for all items *i* and all latent classes *c*. Instead of estimating $K_{i}+1$ probabilities for item *i* and class *c*, $K_{i}$ free parameters $\gamma_{ihc}$ have to be estimated. If all polytomous items have K+1 categories, the multidimensional contingency table of observations has (*K* + 1)*^I^* − 1 free parameters while in the LCM *I* · *K* · *C* + *C* − 1 parameters are estimated. It should be emphasized that the LCM for polytomous items has more free parameters compared to LCMs with dichotomous items as well as for unidimensional and multidimensional item response models for polytomous data.

Like for dichotomous data, restricted LCMs were formulated that represented the vector of all item response functions by $\gamma_{ikc}=q_{ikc}\alpha$ $\left(i=1,\ldots ,I;k=1,\ldots ,K_{i};c=1,\ldots ,C\right)$ using a parameter vector α and known weight vectors $q_{ikc}$ (Formann 1992). It is advisable to induce some structure on item response functions, especially for polytomous data, because many parameters have to be estimated without any structural assumptions.

## 3. Regularized Latent Class Analysis

As LCMs are exploratory models, interpretation of results could sometimes be challenging. Moreover, in not too large samples, parameter estimation gets instable, and findings are sometimes not generalizable across different samples. Alternatively, confirmatory latent models could be estimated for obtaining more stable and more interpretable parameter estimates. However, such confirmatory approaches need assumptions that have to be known in advance of the data analysis. Hence, alternative approaches are sought.

Regularized latent class models (RLCMs; Chen et al. 2017) estimate item response probabilities under the presupposition that similar item response probabilities in these models are grouped and receive the same value. The main idea of using the regularization technique (see Hastie et al. 2015 for an overview) to LCMs is that by subtracting an appropriate penalty term from the log-likelihood function, some simpler structure on item response probabilities is posed. Different penalty terms typically result in different estimated parameter structures. In a recent Psychometrika paper, Chen et al. (2017) proposed the RLCM for dichotomous item responses. Related work for dichotomous data can be found in Wu (2013) and Yamamoto and Hayashi (2015).

The regularization technique has also been applied for factor models with continuous items (Huang et al. 2017; Jacobucci et al. 2016) and discrete items (Chen et al. 2018; Sun et al. 2016) in order to fit exploratory factor models with the goal of estimating as many zero loadings as possible. In this respect, regularization is a viable alternative to factor rotation methods (Scharf and Nestler 2019).

The regularization technique has also been applied to Gaussian mixture models in which cluster means are estimated to be equal for some variables among clusters (Bhattacharya and McNicholas 2014; Ruan et al. 2011). Regularized latent class analysis (RLCA) is also referred to as penalized latent class analysis (see DeSantis et al. 2008). Under this label, LCMs are typically meant by that apply regularization to the estimation of regression coefficients of the latent class regression model (DeSantis et al. 2008, 2012; Houseman et al. 2006; Leoutsakos et al. 2011; Sun et al. 2019; Wu et al. 2018). Fop and Murphy (2018) provide a recent review of applications of the regularization technique in mixture models.

In the following, we describe the RLCM at first for dichotomous items. Afterward, we consider the more complex case of polytomous items in which more possibilities for setting equality constraints among item response probabilities are present.

### 3.1. Regularized Latent Class Analysis for Dichotomous Item Responses

**Figure 1:**
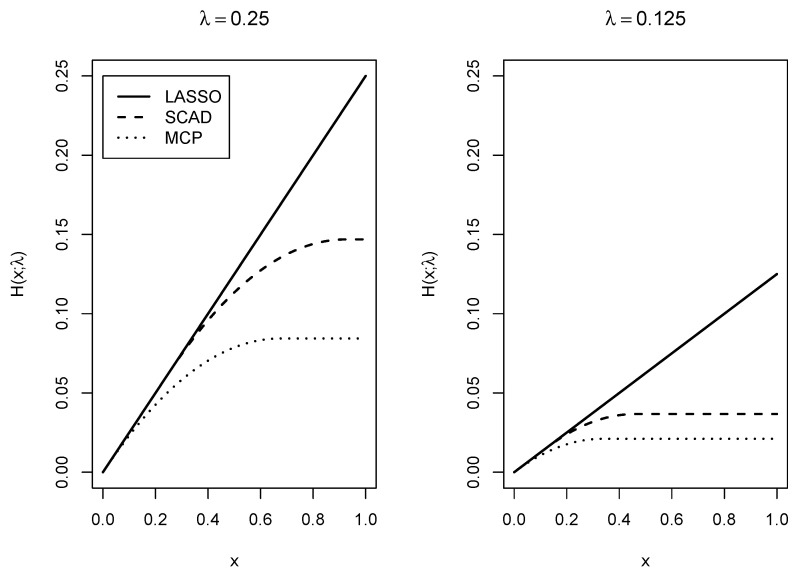
Different penalty functions used in regularization with regularization parameter λ=0.25 (**left panel**) and λ=0.125 (**right panel**).

At first, we consider the case of dichotomous items $X_{i}$ (i=1,…,I). In an RLCM, not all item response probabilities $p_{ic}$ (c=1,…,C) are assumed to be unique. Chen et al. (2017) subtracted a penalty term from the log-likelihood function that penalizes differences in ordered item response probabilities. In more detail, denote by $p_{i,(c)}$ (c=1,…,C) ordered item response probabilities of the original probabilities $p_{ic}$ such that $p_{i,(1)}\leq p_{i,(2)}\leq \ldots p_{i,(C)}$, and collect all parameters in $p_{i}^{*}$. Then, Chen and colleagues used the following penalty function for item *i*

(6) $$Pen\left(p_{i}^{*};\lambda \right)=\sum_{c=2}^{C}H_{\mathrm{SCAD}}\left(p_{i,(c)}-p_{i,(c-1)};\lambda \right),$$

where $H_{\mathrm{SCAD}}$ denotes the smoothly clipped absolute deviation penalty (SCAD; Fan and Li 2001). The SCAD penalty takes a value of zero if $p_{i,(c)}-p_{i,(c-1)}=0$ and is positive otherwise (see Figure 1 for the functional form of the SCAD penalty). The parameter λ is a regularization parameter that governs the strength of the penalty function. With small values of λ, differences are barely penalized, but with large values of λ, differences are heavily penalized, and item parameters approach a uniform distribution.

If X denotes the matrix of observed data and $p^{*}$ denotes the vector of all ordered item response probability and δ the vector that represents the skill class probabilities, the following function is maximized in Chen et al. (2017):

(7) $$l\left(p^{*},\delta ;X\right)-N\sum_{i=1}^{I}Pen\left(p_{i}^{*};\lambda \right),$$

where *l* denotes the log-likelihood function of the data. By employing a penalty function Pen in the estimation, some item response probabilities are merged, which, in turn, eases the interpretation of resulting latent classes. It should be noted that for estimating model parameters, the regularization parameter λ has to be fixed. In practice, the regularization parameter λ also has to be estimated. Hence, the maximization is performed on a grid of λ values (say, λ=0.01,0.02,…,0.30), and that model is selected that is optimal with respect to some criterion. Typical criteria are the cross-validated log-likelihood or information criteria like the Akaike information criterion (AIC), Bayesian information criterion (BIC), or others (Hastie et al. 2015).

The maximization of (7) is conducted using an expectation-maximization (EM) algorithm (see Section 3.3 for general description). The estimation approach of Chen et al. (2017) is implemented in the R package CDM (George et al. 2016; Robitzsch and George 2019).

#### 3.1.1. Fused Regularization among Latent Classes

Though the estimation approach of Chen et al. (2017) is successful, it is not clear how it could be generalized to polytomous data because it is not evident how item response probabilities of several categories should be ordered. Hence, we propose a different estimation approach. We apply the technique of fused regularization (Tibshirani et al. 2005; Tutz and Gertheiss 2016) that penalizes all pairwise differences of item response probabilities. In more detail, for a vector $p_{i}$ of item response probabilities, we replace the penalty (used in Equation (6)) of Chen et al. (2017) by

(8) $$Pen\left(p_{i};\lambda \right)=\sum_{c<d}H_{\mathrm{MCP}}\left(p_{ic}-p_{id};\lambda \right),$$

where $p_{ic}=P\left(X_{i}=1|U=c\right)$ are class-specific item response probabilities, and $h_{\mathrm{MCP}}$ denotes the minimax concave penalty (MCP; Zhang 2010). We do not suppose dramatic differences to the SCAD penalty, but we would expect less biased estimators than using the often employed least absolute shrinkage and selection operator (LASSO) penalty $H_{\mathrm{LASSO}}\left(x;\lambda \right)=\lambda \left|x\right|$ (see Hastie et al. 2015). By using pairwise differences in Equation (8), item response probabilities are essentially merged into item-specific clusters of values that are equal within each cluster. Hence, the same goal as in Chen et al. (2017) is achieved. As explained in Section 2.2, our estimation approach uses transformed item response probabilities γ. Therefore, in the estimation, we replace Equation (8) by

(9) $$Pen\left(\gamma_{i};\lambda \right)=\sum_{c<d}H_{\mathrm{MCP}}\left(\gamma_{ic}-\gamma_{id};\lambda \right).$$

Note that by using the penalty on $\gamma_{i}$ in Equation (9) instead of on $p_{i}$ in Equation (8), a different metric in quantifying differences in item parameters is introduced. By using $\gamma_{i}$, differences in extreme probabilities (i.e., probabilities near 0 or 1) appear to be less similar than by using untransformed probabilities as in (8).

In Figure 1, the LASSO, MCP, and SCAD penalty functions are depicted. It can be seen for *x* values near to 0, the MCP and the SCAD penalty equal the LASSO penalty (i.e., f(x)=λ|x|). For sufficiently large *x* values MCP and SCAD reach an upper asymptote, which is not the case for the LASSO penalty. Hence, for the MCP and SCAD penalty, the penalty is relatively constant for large values of *x*. This property explains why the MCP and SCAD penalty typically results in less biased estimates. It should be noted that the application of the regularization presupposes some sparse structure in the data for obtaining unbiased estimates. In other words, the true data generating mechanism consists of a sufficiently large number of equal item parameters. If all item response probabilities would be different in the data generating model, employing a penalty that forces many item parameters to be equal to each other would conflict the data generating model.

#### 3.1.2. Hierarchies in Latent Class Models

**Figure 2:**
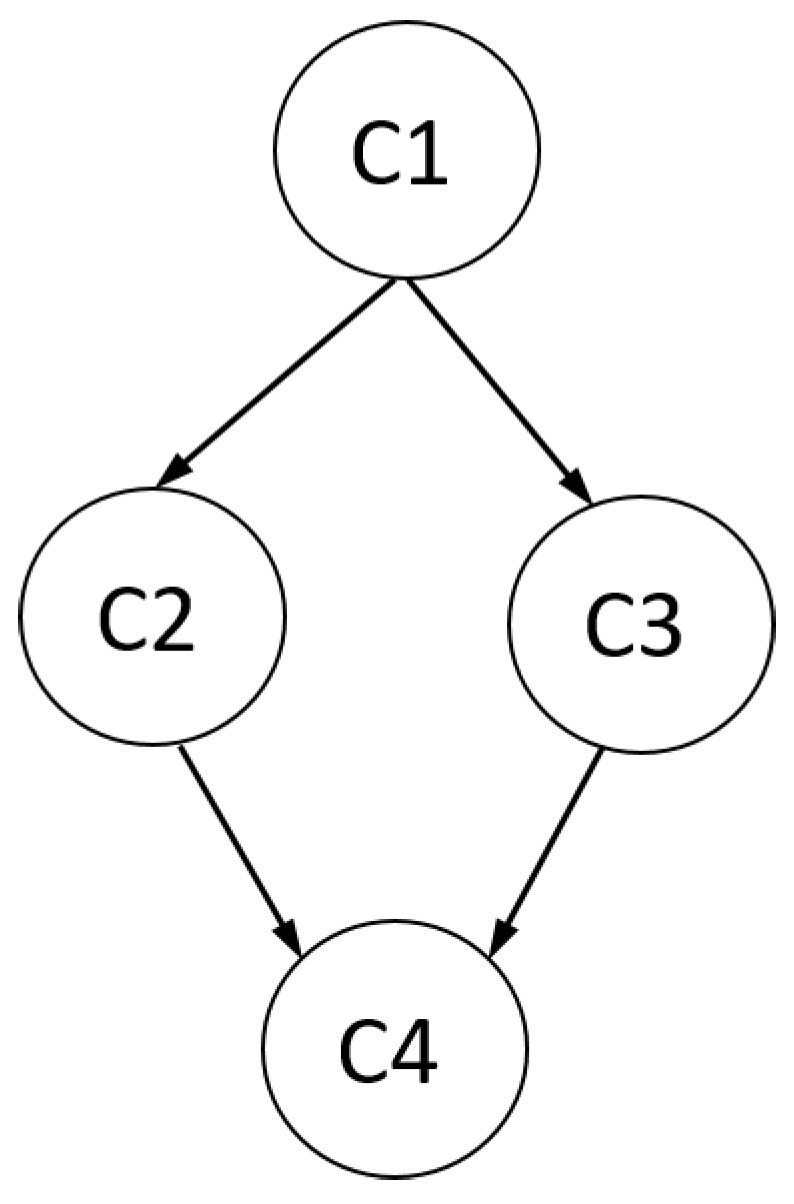
Illustration of a partial order with four latent classes.

The RLCM can be used to derive a hierarchy among latent classes. The main idea is depicted in Figure 2. In an RLCM with C=4 classes, a partial order of latent classes is defined. Class 1 is smaller than Classes 2, 3, and 4. Classes 2 and 3 cannot be ordered. Finally, Classes 2 and 3 are smaller than Class 4. More formally, in an RLCM, we define class *c* to be *smaller* than class *d* (or: class *d* is *larger* than class *c*) if all item response probabilities in class *c* are at most as large as in class *d*, i.e., $p_{ic}\leq p_{id}$ for all items i=1,…,I. We use the notation c⪯d to indicate that *c* is smaller than *d*. In a test with many items, fulfilling these inequalities for all items might be a too strong requirement. Hence, one weakens the concept of partial ordering a bit. Given a tolerable for at most ι items, we say that class *c* is *approximately smaller* than class *d* if $p_{ic}\leq p_{id}$ is fulfilled for at least I−ι items.

The partial ordering of latent classes substantially eases the interpretation of the results in RLCMs. Chen et al. (2017) used the RLCM to derive partially ordered latent classes in cognitive diagnostic modeling. Wang and Lu (2020) also applied the RLCM for estimating hierarchies among latent classes (see also Robitzsch and George 2019). Using the RLCM with an analysis of hierarchies may be considered as a preceding method of confirmatory approaches to latent class modeling.

### 3.2. Regularized Latent Class Analysis for Polytomous Item Responses

In the following, we propose an extension of RLCM for polytomous item responses. It has been shown that using information from item distractors (Myszkowski and Storme 2018; Storme et al. 2019) could increase the reliability for person ability estimates compared to using only dichotomous item responses that only distinguishes between correct and incorrect item responses. Moreover, it could be beneficial to learn about the differential behavior of item distractors analyzing the data based on correct and all incorrect item responses.

Assume that 0 denotes the category that refers to a correct response and $1,\ldots ,K_{i}$ refer to the categories of the distractors. In our parameterization of the LCM for polytomous data (see Section 2.2), only parameters $\gamma_{ikc}$ of distractors *k* for item *i* in classes *c* are parameterized. Given the relatively small sample size of the SPM-LS application data (i.e., N=499), the number of estimated parameters in an unrestricted LCM turn out to be quite large because there are seven distractors per item. Moreover, it could be supposed that the distractors of an item behave similarly. Hence, it would make sense to estimate some item parameters to be equal to each other.

We now outline alternatives for structural assumptions on item response probabilities. Let us fix item *i*. For $K_{i}+1$ categories and *C* classes, $K_{i}\cdot C$ item parameters are modeling (omitting the category 0). Hence, we can distinguish between different strategies to the setting of equalities of item parameters. First, for a fixed category *k*, one can merge some item response probabilities among classes. This means that some of the differences $\gamma_{ikc}-\gamma_{ikd}$ (c≠d) are zero. Hence, a penalty on differences $\gamma_{ikc}-\gamma_{ikd}$ has to be posed. This is just the penalty as for dichotomous items (see Equation (8)), but the regularization is applied for $K_{i}$ categories instead of one category. Second, for a fixed class *c*, some item response probabilities among categories could be merged. In this case, one would impose a penalty on differences $\gamma_{ikc}-\gamma_{ihc}$ (k≠h). Third, penalization among classes and among categories can be simultaneously applied. In the remainder, we outline the different strategies in more detail.

#### 3.2.1. Fused Regularization among Latent Classes

Let $\gamma_{ik*}=\left(\gamma_{ik1},\ldots ,\gamma_{ikC}\right)$ denote the vector of item parameters for item *i* in category *k*. Again, let $\gamma_{i}$ denote the vector of all item parameters of item *i*. For a regularization parameter $\lambda_{1}$ and item *i*, we define the penalty

(10) $$Pen\left(\gamma_{i};\lambda_{1}\right)=\sum_{k=1}^{K_{i}}Pen\left(\gamma_{ik*};\lambda_{1}\right)=\sum_{k=1}^{K_{i}}\sum_{c<d}H_{\mathrm{MCP}}\left(\gamma_{ikc}-\gamma_{ikd};\lambda_{1}\right).$$

As a result, for a category, some item response probabilities will be merged across latent classes. However, the merging of item parameters (also referred to as fusing; Tibshirani et al. 2005) is independently applied for all categories of an item. In practice, it is maybe not plausible that all distractors of an item would function differently, and item parameters should be more regularized.

#### 3.2.2. Fused Regularization among Categories

As a second alternative, we now merge categories. Let $\gamma_{i*c}=\left(\gamma_{i1c},\ldots ,\gamma_{iK_{i}c}\right)$ denote the vector of item parameters for item *i* in class *c*. For a regularization parameter $\lambda_{2}$ and item *i*, we define the penalty

(11) $$Pen\left(\gamma_{i};\lambda_{2}\right)=\sum_{c=1}^{C}Pen\left(\gamma_{i*c};\lambda_{2}\right)=\sum_{c=1}^{C}\sum_{k<h}H_{\mathrm{MCP}}\left(\gamma_{ikc}-\gamma_{ihc};\lambda_{2}\right).$$

As a result, some of the item response probabilities of categories are set equal to each other. As an outcome of applying this penalty, atypical distractors could be detected. However, by using the penalty in Equation (11), no equalities among latent classes are imposed.

#### 3.2.3. Fused Regularization among Latent Classes and Categories

The apparent idea is to combine the regularization among latent classes and categories. By doing so, the penalties in Equations (10) and (11) have to be added. In more detail, for regularization parameters $\lambda_{1}$ and $\lambda_{2}$, we use the penalty

(12) $$Pen\left(\gamma_{i};\lambda_{1},\lambda_{2}\right)=\sum_{k=1}^{K_{i}}\sum_{c<d}H_{\mathrm{MCP}}\left(\gamma_{ikc}-\gamma_{ikd};\lambda_{1}\right)+\sum_{c=1}^{C}\sum_{k<h}H_{\mathrm{MCP}}\left(\gamma_{ikc}-\gamma_{ihc};\lambda_{2}\right).$$

It can be seen that the penalty in Equation (12) now depends on two regularization parameters. In the estimation, the one-dimensional grid of regularization parameters has then to be substituted by a two-dimensional grid. This substantially increases the computational demand.

#### 3.2.4. Fused Group Regularization among Categories

We can now proceed to pose additional structural assumptions on item parameters. One could suppose that two distractors *k* and *h* of item *i* show the same behavior. In the RLCM, this means that $\gamma_{ikc}-\gamma_{ihc}=0$ holds for all classes c=1,…,C. The group regularization technique allows us to estimate all parameters in a subset of parameters to be zero (see Huang et al. 2012 for a review). A fused group regularization approach presupposes that either all differences $\gamma_{ikc}-\gamma_{ihc}$ equal zero or all differences are estimated to be different from zero (Cao et al. 2018; Liu et al. 2019). This property can be achieved by substituting a norm of the difference of the two vectors in the penalty. In more detail, one considers the penalty

(13) $$Pen\left(\gamma_{i};\lambda_{1}\right)=\sum_{k<h}H_{\mathrm{MCP}}\left(|\right|\gamma_{ik*}-\gamma_{ih*}||;\lambda_{1})$$

where for a vector $x=(x_{1},\ldots ,x_{p})$, the norm ||x|| is defined as $\left||x|\right|=\sqrt{p}\sqrt{\sum_{k=1}^{p}x_{k}^{2}}$. In practice, using the penalty in Equation (13) could provide a more parsimonious estimation than the penalty defined in Equation (12). In principle, model comparisons can be carried out to decide which assumption is better represented in the data.

#### 3.2.5. Fused Group Regularization among Classes

Alternatively, one could also assume that latent classes function the same among classes. In the RLCM, then it would hold that that $\gamma_{ikc}-\gamma_{ikd}=0$ for all categories $k=1,\ldots ,K_{i}$. A fused group regularization results in the property that either all item parameters of classes *c* and *d* are equal to each other or all estimated to be different from each other. The following penalty is used in this case:

(14) $$Pen\left(\gamma_{i};\lambda_{2}\right)=\sum_{c<d}H_{\mathrm{MCP}}\left(|\right|\gamma_{i*c}-\gamma_{i*d}||;\lambda_{2})$$

### 3.3. Estimation

We now describe the estimation of the proposed RLCM for polytomous data. Let $X=(x_{nik})$ denote the observed dataset where $x_{nik}$ equals 1 if person *n* (n=1,…,N) chooses category *k* for item *i*. Let $\gamma_{i}$ denote item parameters of item *i* and and the vector that contains item parameters of all items. The vector δ represents the skill class distribution. Furthermore, let *p_ic_*(*x*; ***γ**_i_*) = *P*(*X_i_* = *x* | *U* = *c*) and *p_c_*(***δ***) = *P*(*U* = *c*).

Following Chen et al. (2017) and Sun et al. (2016), an EM algorithm is applied for estimating model parameters. The complete-data log-likelihood function is given

(15) $$l_{\mathrm{com}}\left(\gamma ,\delta ,U\right)=\sum_{n=1}^{N}\sum_{i=1}^{I}\sum_{k=1}^{K_{i}}\sum_{c=1}^{C}x_{nik}u_{nc}\log p_{ic}\left(k;\gamma_{i}\right)+\sum_{n=1}^{N}\sum_{c=1}^{C}u_{nc}\log p_{c}\left(\delta \right),$$

where $u_{n}=\left(u_{n1},\ldots ,u_{nC}\right)$ is the vector of latent class indicators for person *n*. It holds that $u_{nc}=1$ if person *n* is located in class *c*. Obviously, the true class membership is unknown and, hence, Equation (15) cannot be used for maximization.

In the EM algorithm, the estimation of $l_{\mathrm{com}}$ is replaced by the expected complete-data log-likelihood function by integrating over the posterior distribution. In more detail, unobserved values $u_{nc}$ are replaced by their conditional expectations:

(16) $$u_{nc}^{*}=E\left(u_{nc}|x_{n};\gamma^{\left(t\right)},\delta^{\left(t\right)}\right)=\frac{p_{c}\left(\delta^{\left(t\right)}\right)\prod_{i=1}^{I}\prod_{k=1}^{K_{i}}p_{ic}{\left(k;\gamma_{i}^{\left(t\right)}\right)}^{x_{nki}}}{\sum_{d=1}^{D}p_{d}\left(\delta^{\left(t\right)}\right)\prod_{i=1}^{I}\prod_{k=1}^{K_{i}}p_{id}{\left(k;\gamma_{i}^{\left(t\right)}\right)}^{x_{nki}}}\;\left(c=1,\ldots ,C\right),$$

where $\gamma^{\left(t\right)}$ and $\delta^{\left(t\right)}$ are parameter estimates from a previous iteration *t*. The EM algorithm alternates between the E-step and the M-step. By replacing the unobserved values $u_{ni}$ by their expected values $u_{ni}^{*}$, the following *Q*-function is obtained that is used for maximization in the M-step

(17) $$Q\left(\gamma ,\delta |\gamma^{\left(t\right)},\delta^{\left(t\right)}\right)=\sum_{n=1}^{N}\sum_{i=1}^{I}\sum_{k=1}^{K_{i}}\sum_{c=1}^{C}x_{nik}u_{nc}^{*}\log p_{ic}\left(k;\gamma_{i}\right)+\sum_{n=1}^{N}\sum_{c=1}^{C}u_{nc}^{*}\log p_{c}\left(\delta \right).$$

From this *Q*-function, the penalty function is subtracted such that the following function is minimized for some regularization parameter λ in the M-step

(18) $$Q\left(\gamma ,\delta |\gamma^{\left(t\right)},\delta^{\left(t\right)}\right)-N\sum_{i=1}^{I}Pen\left(\gamma_{i};\lambda \right).$$

It can be seen that item parameters $\gamma_{i}$ are separately obtained for each item *i* in the M-step because the penalties are defined independently for each item. Hence, for each item *i*, one maximizes

(19) $$\sum_{n=1}^{N}\sum_{k=1}^{K_{i}}\sum_{c=1}^{C}x_{nik}u_{nc}^{*}\log p_{ic}\left(k;\gamma_{i}\right)-NPen\left(\gamma_{i};\lambda \right).$$

Latent class probability parameters δ are also obtained independently from item parameters in the M-step.

The penalty function Pen turns out to be non-differentiable. Here, we use a differentiable approximation of the penalty function (Oelker and Tutz 2017; see also Battauz 2019). As it is well known that the log-likelihood function in LCMs is prone to multiple maxima, using multiple starting values in the estimation is advised.

The described EM algorithm is included in an experimental version of the function regpolca() in the R package sirt (Robitzsch 2020). The function is under current development for improving computational efficiency.

## 4. Simulated Data Illustration

Before we illustrate the application of the method to the SPM-LS dataset, we demonstrate the technique using a simulated data set. This helps to better understand the proposed method of regularized latent class modeling under ideal conditions.

### 4.1. Dichotomous Item Responses

#### 4.1.1. Data Generation

First, we consider the case of dichotomous items. To mimic the situation in the SPM-LS dataset, we also chose I=12 items for simulating a dataset. Moreover, to reduce sampling uncertainty somewhat, a sample size of N=1000 subjects was chosen. There were C=4 latent classes with true class probabilities 0.30, 0.20, 0.10, and 0.40. In Table 1, we present the item response probabilities with each cluster. We only specified parameters for six items and duplicated these parameters for the remaining six items in the test. It can be seen in Table 1 that many item response probabilities were set equal to each other. Indeed, for the first four items, there are only two instead of four unique probabilities. Moreover, it is evident from Table 1 that the four classes are partially ordered. The first class has the lowest probabilities for all items and is, therefore, the smallest class that consists of the least proficient subjects. The fourth class has the highest probabilities, constitutes the largest class, and contains the most proficient subjects.

The model selection is carried out using information criteria AIC and BIC. For regularized models, the required number of parameters in the computation of information criteria is determined by the number of estimated unique parameters. For example, if four item response probabilities would be estimated to be equal in a model, only one parameter would be counted.

#### 4.1.2. Results

In the first step, we estimated exploratory latent class models with C=2, 3, 4, 5, and 6 classes. The model comparison is presented in Table 2. While the decision based on the AIC was ambiguous and selected the incorrect number of classes, the BIC correctly selected model with C=4 latent classes. This observation is consistent with the literature that argues that model selection in LCMs should be based on the BIC instead of the AIC (Collins and Lanza 2009; Keribin 2000).

In the solution with four classes, estimated class probabilities were 0.290, 0.204, 0.120, and 0.386, respectively, which closely resembled the data generating values. In Table 3, estimated item response probabilities are shown. The estimates were very similar to the data generating parameters that are presented in Table 1. It can be seen that some deviations from the simulated equality constraints are obtained. It is important to emphasize that latent class solutions are not invariant with respect to their class labels (so-called label switching). Class labels in the estimated model have to be permuted in order to match the class label in the simulated data.

Finally, we estimated the RLCM for regularization parameters from 0.01 to 1.00 in steps of 0.01 for C=4 classes in order to obtain the best-fitting solution. The regularization parameter λ=0.21 provided the best-fitting model in terms of the BIC (BIC= 13,104, AIC= 12,957). Notably, this model showed a substantially better model fit than the exploratory LCM with four classes due to the more parsimonious estimation of item parameters. In the model with λ=0.21, in total, 21 item parameters were regularized (i.e., they were set equal to item parameters in other classes for the respective item), resulting in 30 freely estimated model parameters. With respect to the AIC, the best-fitting model was obtained for λ=0.15 (BIC= 13,110, AIC= 12,953). This model resulted in 19 regularized item parameters and 32 freely estimated item parameters. The model selected by the minimal BIC (λ=0.21) resulted in estimated class probabilities of 0.29, 0.21, 0.11, and 0.39. Estimated item response probabilities (shown in Table 4) demonstrate that the equality constraints that were posed in the data generating were correctly identified in the estimated model.

**Figure 3:**
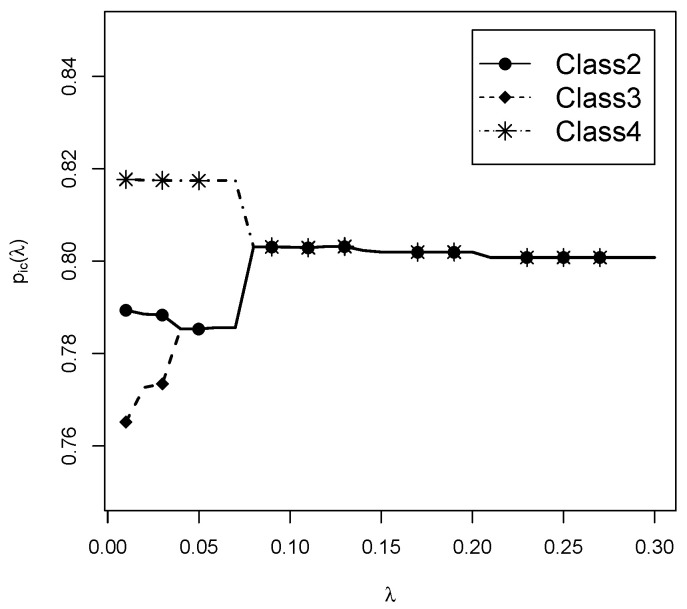
Data illustration dichotomous data: Regularization path for estimated item response probabilities for Item 1 for Classes 2, 3, 4 for the four-class solution.

Lastly, we want to illustrate the behavior of regularization. For the sequence of specified regularization parameters λ in the estimation, the estimated item response probabilities $p_{ic}\left(\lambda \right)$ can be plotted. Such a plot is also referred to as a regularization path (Hastie et al. 2015). With very small λ values, no classes were merged, and all item parameters were estimated differently from each other. With increasing values of λ, item parameters were subsequently merged. For Item 1 and Classes 2, 3, and 4, the regularization path is shown in Figure 3. At first, item parameters of Class 2 and 4 are merged at λ=0.04. Afterwards, all three item response probabilities are merged at λ=0.09.

### 4.2. Polytomous Item Responses

#### 4.2.1. Data Generation

Now, we simulate illustrated data with polytomous item responses with 12 items, each item possessing four categories (i.e., $K_{i}=3$). The first category (i.e., Category 0) refers to the correct category, while categories 1, 2, and 3 refer to distractors of the item. As in the case of dichotomous data, item response probabilities were 0.30, 0.20, 0.10, and 0.40, and N=1000 subjects were simulated. Again, we specified item parameters for the first six items and replicated the parameters for the remaining six items. In Table 5, true item response probabilities are shown that were used for generating the dataset. It is evident that the item response probabilities are strongly structured. All distractors of Items 1 and 5 function precisely the same. For Item 2, Category 1, and Category 2 show the same behavior. Category 3 only shows a differential behavior in Classes 2 and 4. At the other extreme, all item response probabilities differ for Item 6 among classes and categories. It can be expected that an RLCM will result in a substantial model improvement compared to an exploratory LCM without equality constraints.

#### 4.2.2. Results

At first, we fitted exploratory LCMs with 2, 3, 4, 5, and 6 classes. Based on the information criteria presented in Table 6, the correct model with C=4 latent classes was selected. However, the difference in model improvement by moving from 3 to 4 classes would be considered as negligible (i.e., a BIC difference of 3) in practice. Estimated latent class probabilities in the model with four latent classes were estimated as 0.29, 0.21, 0.12, and 0.38. Estimated item response probabilities are shown in Table A1 in Appendix A.

In the next step, different RLCMs for polytomous data were specified. As explained in Section 3.2, one can regularize differences in item parameters among classes (using a regularization parameter $\lambda_{1}$), among categories (using a regularization parameter $\lambda_{2}$), or both (using both regularization parameters or applying fused group regularization). We fitted the five approaches (Approaches R1, *…*, R5) that were introduced in Section 3.2 to the simulated data using unidimensional and two-dimensional grids of regularization parameters. For each of the regularization approaches, we selected the model with minimal BIC.

In Table 7, it can be seen that the model with the fused penalty on item categories fitted the model best (Approach R2: BIC= 24,689). In this model, 79 item parameters are regularized. The decrease in BIC compared to an exploratory LCM is substantial. From these findings, it follows for this dataset that it is important to fuse item parameters among categories instead of among classes. The best-fitting model when using a simultaneous penalty for classes and categories (Approach R3: BIC= 24,836) outperformed the model in which only parameters were fused among classes (Approach R1: BIC= 24,932). However, it was inferior to the model with fusing among categories. Notably, the largest number of regularized parameters (#nreg=103) was obtained for Approach R3. The fused grouped regularization approaches (Approaches R4 and R5) also improved fit compared to an unrestricted exploratory LCM but were also inferior to R2. The reason might be that applying group regularization results in the extreme decision that either all item parameters are equal or all are different. In contrast, fused regularization Approaches R1, R2, and R3 allow the situation in which only some of the item parameters are estimated to be equal to each other.

For the best-fitting model of Approach R2 (i.e., fusing among categories), estimated class probabilities were 0.29, 0.22, 0.10, and 0.39, respectively. Estimated item response probabilities from this model are shown in Table 8. It can be seen that model estimation was quite successful in identifying parameters that were equal in the data generating model.

## 5. Application of the SPM-LS Data

In this section, we illustrate the use of RLCM to the SPM-LS dataset.

### 5.1. Method

According to the topic of this special issue, the publicly available dataset from the Myszkowski and Storme (2018) study was reanalyzed. The original study compared various parametric item response models (i.e., 1PL, 2PL, 3PL, 4PL, and nested logit model) performed on a dataset comprised of N=499 students (214 males and 285 females) aged between 19 and 24. The analyzed data consisted of responses on the 12 most difficult SPM items and are made freely available at https://data.mendeley.com/datasets/h3yhs5gy3w/1. For details regarding the data gathering procedure, we refer to Myszkowski and Storme (2018).

Each of the I=12 items had one correct category and $K_{i}=7$ distractors. To be consistent with the notation introduced in the paper and to ease interpretation of the results, we recoded the original dataset when using polytomous item responses. First, we scored the correct response as Category 0. Second, we recoded the order of distractors according to their frequency. In more detail, Category 1 in our rescored dataset was the most attractive distractor (i.e., most frequent distractor), while Category 7 was the least attractive distractor. The relative frequencies and references to categories of the original dataset are shown in Table 9. It could be supposed that there some especially attractive distractors for each item. However, many item category frequencies turned out to be relatively similar such that it could be that they would also function homogeneously among latent classes. We also analyzed the SPM-LS dataset in its dichotomous version in which Category 1 was scored as correct, and Category 0 summarized responses of all distractors.

For the dataset with dichotomous items, the exploratory LCM and the RLCM were fitted for two to six latent classes. Model selection was conducted based on the BIC. For the dataset with rescored polytomous items, we used the same number of classes for estimating the exploratory LCM. For the RLCM, we applied the fused regularization approach with respect to classes (Section 3.2.1), categories (Section 3.2.2), and to classes and categories in a simultaneous manner (Section 3.2.3).

### 5.2. Results

#### 5.2.1. Results for Dichotomous Item Responses

For the SPM-LS dataset with dichotomous items, a series of exploratory LCMs and RLCMs with two to six classes was fitted. According to the BIC presented in Table 10, an exploratory LCM with four classes would be selected. When RLCMs were fitted, a model with five classes would be selected that had 19 regularized item parameters.

**Figure 4:**
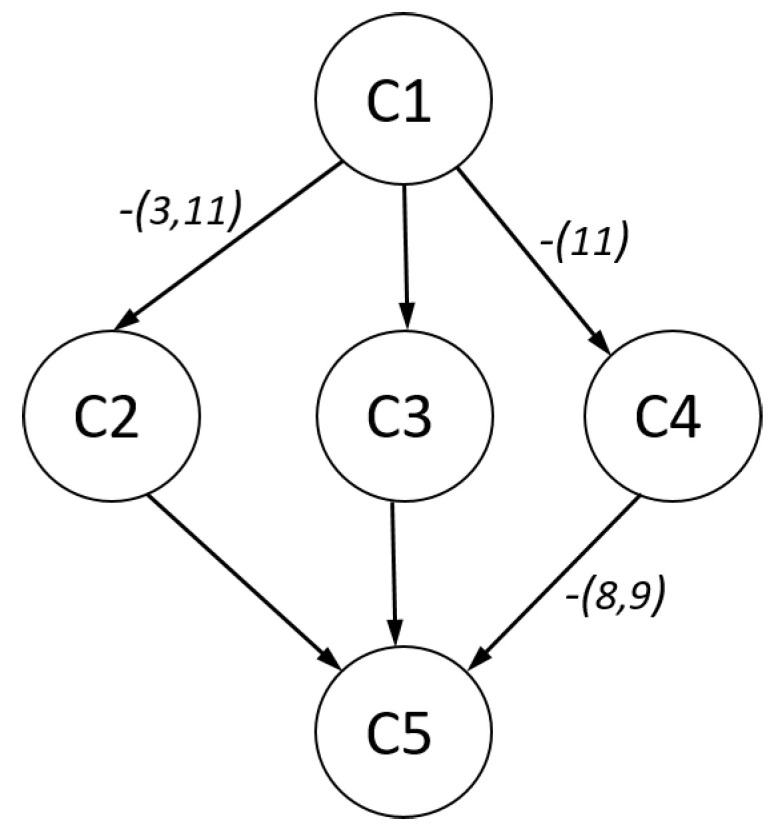
SPM-LS dichotomous data: partial order for latent class from RLCM.

In Table 11, item response probabilities and skill class probabilities for the RLCM with C=5 classes are shown. By considering the average item response probabilities per skill class ${\bar{p}}_{•c}=\left(\sum_{i=1}^{I}p_{ic}\right)/I$, Class C1 (12% frequency) was the least performing and Class C5 (37% frequency) the best performing class. Class C3 (40% frequency) could be seen as an intermediate class. Classes C2 and C4 were relatively rare. Compared to the medium Class C3, students in Class C2 had a particularly bad performance at Items 3, 6, and 11, but outperformed them on Items 7, 8, and 12. Students in Class C4 showed perfect performance on Items 8 and 9, but notably worse performance on Items 10 and 11. Interestingly, one could define a partial order on the classes if we allowed at most two violations of inequality conditions. In Figure 4, this partial order is depicted. The arrow from Class C1 to Class C3 means that C1 was smaller than C3. There are arrows with particular labels that indicate violations of the partial order. For example, C1 was approximately smaller than C2, and Items 3 and 11 violated the ordering property. To summarize, three out of the five classes fulfilled the ordering property for all items. Two classes possessed violations for two or three items and could be interpreted to detect subpopulations of subjects that showed latent differential item functioning.

#### 5.2.2. Results for Polytomous Item Responses

We now only briefly discuss the findings for the analysis of the SPM-LS dataset based on polytomous item responses. For the exploratory latent class models, the model with just two latent classes would be selected according to the BIC. However, the model with six latent classes would be selected according to the AIC. Given a large number of estimated item parameters, applying the RLCM seems to be required for obtaining a parsimonious model. The best-fitting model was obtained with C=3 classes by fusing categories with a regularization parameter of $\lambda_{2}=0.24$. Classes C1 (28% frequency) and C2 (5% frequency) had low performance, while Class C3 was the high-performing class (67% frequency). As an illustration, we provide in Table 12 estimated item probabilities for the last three items. It can be seen that some of the categories were fused such that they had equal item response probabilities within a latent class. All item parameters are shown in Table A2 in Appendix B.

At the time of writing, results for polytomous data for the SPM-LS do not seem to be very consistent with those for dichotomous data. It could be the large number of parameters to be estimated (several hundred depending on the number of classes) for the relatively small sample size of N=499 is critical. Other research has also shown that regularization methods for LCMs need sample sizes of at least 1000 or even more for performing satisfactorily (Chen et al. 2015).

## 6. Discussion

In this article, we proposed an extension of regularized latent class analysis to polytomous item responses. We have shown using the simulated data illustration and the SPM-LS dataset that fusing among classes or categories can be beneficial in terms of model parsimony and interpretation. Often, conceptualizing substantive questions as latent classes led researchers to easier to think in types of persons. This interpretation is not apparent in latent variables with continuous latent variables.

In our regularization approach to polytomous data, we based regularization penalties on distractors of items. Hence, the correct item response serves as a reference category. In LCA applications in which the definition of a reference category cannot be done, the regularization approach has certainly to be modified. Note that for $K_{i}+1$ categories, only $K_{i}$ item parameters per class can be independently estimated. Alternatively, a sum constraint $\sum_{k=0}^{K_{i}}\gamma_{ikc}=0$ could be posed if $\gamma_{ikc}$ ($k=0,1,\ldots ,K_{i}$ denotes the item parameters of item *i* of category *k* in class *c*. Such constraints can be replaced by adding ridge-type penalties of the form $\lambda_{3}\sum_{k=0}^{K_{i}}\gamma_{ikc}^{2}$ to the fused regularization penalty, where $\lambda_{3}$ is another regularization parameter. By squaring item parameters in the penalty function, they are uniformly shrunk to zero in the estimation.

By treating the correct item response as the reference category, regularization only operates on the categories for the incorrect response. As pointed out by an anonymous reviewer, it could be more appropriate by fusing classes for the correct item response and for incorrect item response categories separately. This would lead to an overidentified model because all class-specific item response probabilities would appear in the model. However, if, again, a ridge-type would be employed, the identification issue would disappear.

As the application of the regularization technique to an LCM results in a particular restricted LCM, it has to be shown that the model parameters can be identified. The analysis of necessary and sufficient conditions for identification in restricted LCMs was currently investigated (Gu and Xu 2018; Xu 2017). Because the inclusion of the penalty function, accompanied by a regularization parameter, introduces an additional amount of information in the estimation, it is unclear whether identifiability should be studied only on the likelihood part of the optimization function (see San Martín 2018 for a related discussion in Bayesian estimation). 

It should be noted that similar regularization approaches have been studied for cognitive diagnostic models (Chen et al. 2015; Gu and Xu 2019; Liu and Kang 2019; Xu and Shang 2018). These kinds of models pose measurement models on *D* dichotomous latent variables. These *D* latent variables constitute $2^{D}$ latent classes. In addition, in this model class, the modeling of violations of the local independence assumption in LCA has been of interest (Kang et al. 2017; Tamhane et al. 2010).

Previous articles on the SPM-LS dataset also used distractor information by employing the nested logit model (NLM; Myszkowski and Storme 2018). The NLM is also very data-hungry, given the low sample size of the dataset. It has been argued that reliability can be increased by using distractor information (Myszkowski and Storme 2018; Storme et al. 2019). It should be noted that this is only true to the extent that item parameters can be reliably estimated. For N=499 in the SPM-LS dataset, this will probably be not the case. Regularized estimation approaches could circumvent estimation issues (see Battauz 2019 for a similar approach in the nominal response model).

Finally, we would like to emphasize that the regularization approaches can be interpreted as empirical Bayesian approaches that employ hierarchical prior distributions on item parameters (van Erp et al. 2019). It can be expected that Bayesian variants of RLCMs are competitive to EM-based estimation, especially for small(er) samples.

## Figures and Tables

**Table 1:** Data illustration dichotomous data: true item response probabilities $p_{ic}$.

Item	Class			
	1	2	3	4
1, 7	0.10	0.82	0.82	0.82
2, 8	0.22	0.88	0.88	0.88
3, 9	0.16	0.79	0.16	0.79
4, 10	0.25	0.85	0.25	0.85
5, 11	0.10	0.10	0.46	0.91
6, 12	0.22	0.22	0.22	0.79

**Table 2:** Data illustration dichotomous data: model comparison for exploratory latent class models (LCMs).

*C*	#np	AIC	BIC
2	25	13,636	13,759
3	38	13,169	13,356
4	51	12,979	**13,229**
5	64	12,981	13,295
6	76	**12,976**	13,349

*Note:C* = number of classes; #np = number of estimated parameters.

**Table 3:** Data illustration dichotomous data: estimated item response probabilities in exploratory LCM with C=4 classes.

Item	Class			
	1	2	3	4
1	0.08	0.79	0.79	0.82
2	0.20	0.84	0.89	0.91
3	0.15	0.76	0.19	0.81
4	0.27	0.90	0.29	0.86
5	0.10	0.09	0.44	0.92
6	0.23	0.23	0.30	0.77
7	0.07	0.79	0.82	0.85
8	0.24	0.87	0.91	0.87
9	0.14	0.82	0.18	0.81
10	0.19	0.90	0.42	0.83
11	0.10	0.13	0.54	0.89
12	0.25	0.19	0.19	0.77

**Table 4:** Data illustration dichotomous data: estimated item response probabilities in the regularized latent class model (RLCM) with C=4 classes based on the minimal Bayesian information criterion (BIC) (λ=0.21).

Item	Class			
	1	2	3	4
1	0.08	0.80	0.80	0.80
2	0.20	0.88	0.88	0.88
3	0.15	0.79	0.15	0.79
4	0.27	0.87	0.27	0.87
5	0.09	0.09	0.44	0.92
6	0.23	0.23	0.23	0.76
7	0.07	0.82	0.82	0.82
8	0.24	0.87	0.87	0.87
9	0.14	0.81	0.14	0.81
10	0.19	0.85	0.40	0.85
11	0.11	0.11	0.55	0.89
12	0.22	0.22	0.22	0.76

**Table 5:** Data illustration polytomous data: true item response probabilities $p_{ic}$.

Item	Cat	Class				Item	Cat	Class			
		1	2	3	4			1	2	3	4
1, 7	0	0.10	0.82	0.82	0.82	4, 10	0	0.25	0.85	0.25	0.85
1, 7	1	0.30	0.06	0.06	0.06	4, 10	1	0.35	0.03	0.35	0.03
1, 7	2	0.30	0.06	0.06	0.06	4, 10	2	0.20	0.03	0.20	0.03
1, 7	3	0.30	0.06	0.06	0.06	4, 10	3	0.20	0.09	0.20	0.09
2, 8	0	0.22	0.88	0.88	0.88	5, 11	0	0.10	0.10	0.46	0.91
2, 8	1	0.26	0.05	0.04	0.06	5, 11	1	0.30	0.30	0.18	0.03
2, 8	2	0.26	0.05	0.04	0.06	5, 11	2	0.30	0.30	0.18	0.03
2, 8	3	0.26	0.02	0.04	0.00	5, 11	3	0.30	0.30	0.18	0.03
3, 9	0	0.16	0.79	0.16	0.79	6, 12	0	0.22	0.22	0.22	0.79
3, 9	1	0.28	0.11	0.28	0.11	6, 12	1	0.24	0.23	0.22	0.06
3, 9	2	0.33	0.05	0.33	0.05	6, 12	2	0.20	0.17	0.12	0.04
3, 9	3	0.23	0.05	0.23	0.05	6, 12	3	0.34	0.38	0.44	0.11

**Table 6:** Data illustration polytomous data: model comparison for exploratory LCMs.

*C*	#np	AIC	BIC
2	72	25,082	25,440
3	107	24,616	25,151
4	143	**24,431**	**25,148**
5	179	24,439	25,337
6	215	24,444	25,524

*Note:C* = number of classes; #np = number of estimated parameters.

**Table 7:** Data illustration polytomous data: model comparison for different RLCMs with four classes.

Appr.	Fused	Equation	*C*	$\lambda_{1}$	$\lambda_{2}$	#np	#nreg	BIC
R1	Class	(10)	4	0.31	—	84	63	24,982
R2	Cat	(11)	4	—	0.18	68	79	**24,689**
R3	Cat and Class	(12)	4	0.40	0.15	44	103	24,836
R4	Grouped Cat	(13)	4	0.45	—	82	65	24,777
R5	Grouped Class	(14)	4	0.65	—	79	67	24,776

*Note:* Appr. = approach; Cat = category; Eq. = equation for regularization penalty in Section 3.2; *C* = number of classes; #np = number of estimated parameters; #nreg = number of regularized item parameters.

**Table 8:** Data illustration polytomous data: estimated item response probabilities in the RLCM with C=4 classes and fused regularization among classes based on the minimal BIC.

Item	Cat	Class				Item	Cat	Class				Item	Cat	Class			
		1	2	3	4			1	2	3	4			1	2	3	4
1	0	0.07	0.79	0.82	0.82	5	0	0.10	0.13	0.46	0.92	9	0	0.13	0.82	0.10	0.82
1	1	0.31	0.07	0.06	0.06	5	1	0.30	0.29	0.18	0.02	9	1	0.29	0.06	0.30	0.06
1	2	0.31	0.07	0.06	0.06	5	2	0.30	0.29	0.18	0.02	9	2	0.29	0.06	0.30	0.06
1	3	0.31	0.07	0.06	0.06	5	3	0.30	0.29	0.18	0.04	9	3	0.29	0.06	0.30	0.06
2	0	0.22	0.85	0.87	0.91	6	0	0.22	0.24	0.30	0.76	10	0	0.20	0.89	0.37	0.82
2	1	0.26	0.05	0.01	0.06	6	1	0.26	0.18	0.32	0.07	10	1	0.42	0.05	0.25	0.05
2	2	0.26	0.05	0.01	0.03	6	2	0.26	0.18	0.06	0.03	10	2	0.19	0.01	0.13	0.03
2	3	0.26	0.05	0.11	0.00	6	3	0.26	0.40	0.32	0.14	10	3	0.19	0.05	0.25	0.10
3	0	0.16	0.74	0.19	0.80	7	0	0.07	0.79	0.85	0.85	11	0	0.10	0.16	0.55	0.91
3	1	0.28	0.16	0.27	0.10	7	1	0.31	0.09	0.05	0.05	11	1	0.30	0.28	0.15	0.03
3	2	0.28	0.05	0.27	0.05	7	2	0.31	0.09	0.05	0.05	11	2	0.30	0.28	0.15	0.03
3	3	0.28	0.05	0.27	0.05	7	3	0.31	0.03	0.05	0.05	11	3	0.30	0.28	0.15	0.03
4	0	0.26	0.88	0.28	0.85	8	0	0.25	0.86	0.91	0.88	12	0	0.24	0.20	0.20	0.76
4	1	0.36	0.05	0.24	0.04	8	1	0.25	0.06	0.03	0.06	12	1	0.21	0.21	0.35	0.10
4	2	0.19	0.02	0.24	0.02	8	2	0.25	0.06	0.03	0.06	12	2	0.21	0.21	0.10	0.04
4	3	0.19	0.05	0.24	0.09	8	3	0.25	0.02	0.03	0.00	12	3	0.34	0.38	0.35	0.10

*Note:* Cat = category.

**Table 9:** The last series of Raven’s standard progressive matrices (SPM-LS) polytomous data: percentage frequencies and recoding table.

Item	Cat0	Cat1	Cat2	Cat3	Cat4	Cat5	Cat6	Cat7
SPM1	76.0 (7)	13.6 (3)	3.0 (1)	2.4 (4)	2.2 (6)	2.0 (2)	0.8 (5)	—
SPM2	91.0 (6)	3.0 (3)	2.4 (4)	2.2 (1)	0.8 (5)	0.4 (7)	0.2 (2)	—
SPM3	80.4 (8)	8.0 (2)	4.2 (6)	2.0 (4)	1.8 (3)	1.6 (5)	1.2 (7)	0.8 (1)
SPM4	82.4 (2)	5.6 (3)	3.2 (5)	2.6 (1)	2.2 (8)	1.8 (6)	1.2 (7)	1.0 (4)
SPM5	85.6 (1)	3.8 (2)	3.0 (3)	2.6 (7)	1.8 (6)	1.6 (5)	1.0 (4)	0.6 (8)
SPM6	76.4 (5)	7.0 (4)	5.2 (6)	3.0 (3)	2.8 (7)	2.6 (8)	2.0 (2)	1.0 (1)
SPM7	70.1 (1)	6.6 (4)	5.8 (5)	5.4 (3)	4.4 (8)	3.4 (6)	2.4 (7)	1.8 (2)
SPM8	58.1 (6)	7.6 (1)	7.0 (3)	6.6 (8)	6.4 (2)	6.2 (5)	5.8 (7)	2.2 (4)
SPM9	57.3 (3)	12.0 (5)	9.0 (1)	7.2 (4)	6.6 (8)	4.0 (7)	3.0 (2)	0.8 (6)
SPM10	39.5 (2)	17.2 (6)	11.2 (7)	8.0 (3)	7.8 (8)	7.4 (5)	6.0 (4)	2.8 (1)
SPM11	35.7 (4)	14.0 (1)	13.8 (7)	9.8 (5)	9.4 (6)	8.0 (3)	6.6 (2)	2.6 (8)
SPM12	32.5 (5)	15.4 (2)	14.2 (3)	10.4 (1)	8.2 (4)	8.2 (7)	7.4 (6)	3.6 (8)

*Note:* Numbers in parentheses denote the original item category.

**Table 10:** SPM-LS dichotomous data: model comparison for exploratory LCMs and RLCM.

	*C*	λ	#np	#nreg	BIC
LCM	2	0	25	0	5973
	3	0	38	0	5721
	4	0	51	0	**5680**
	5	0	64	0	5696
	6	0	77	0	5694
RLCM	2	0.01	25	0	5973
	3	0.33	35	3	5715
	4	0.38	39	12	5643
	5	0.29	45	19	**5621**
	6	0.53	45	32	5620

*Note:C* = number of classes; λ = regularization parameter of selected model with minimal BIC; #np = number of estimated parameters; #nreg = number of regularized parameters.

**Table 11:** SPM-LS dichotomous data: estimated item probabilities and latent class probabilities for best fitting RLCM with C=5 latent classes.

Item	Class				
	C1	C2	C3	C4	C5
$p_{c}$	0.12	0.04	0.40	0.07	0.37
SPM1	0.39	0.39	0.83	0.83	0.83
SPM2	0.57	0.57	0.99	0.86	0.99
SPM3	0.33	0.00	0.86	0.96	0.96
SPM4	0.05	1.00	0.91	0.60	1.00
SPM5	0.08	1.00	0.96	0.77	1.00
SPM6	0.07	0.07	0.85	0.85	0.97
SPM7	0.20	0.83	0.58	0.83	0.95
SPM8	0.06	0.69	0.36	1.00	0.90
SPM9	0.16	0.34	0.34	1.00	0.90
SPM10	0.00	0.23	0.23	0.00	0.79
SPM11	0.14	0.00	0.14	0.00	0.77
SPM12	0.11	0.62	0.11	0.11	0.62
${\bar{p}}_{•c}$	0.18	0.48	0.60	0.65	0.89

*Note:*$p_{c}$ = skill class probability; ${\bar{p}}_{•c}$ = average of item probabilities within class *c*.

**Table 12:** SPM-LS polytomous data: estimated item response probabilities and latent class probabilities for best-fitting RLCM with C=3 latent classes for items SPM10, SPM11 and SPM12.

Item	Cat	C1	C2	C3	Item	Cat	C1	C2	C3	Item	Cat	C1	C2	C3
SPM10	0	0.08	0.00	0.56	SPM11	0	0.11	0.26	0.48	SPM12	0	0.03	0.24	0.45
SPM10	1	0.14	0.12	0.19	SPM11	1	0.18	0.43	0.10	SPM12	1	0.14	0.09	0.17
SPM10	2	0.26	0.40	0.03	SPM11	2	0.21	0.00	0.12	SPM12	2	0.19	0.44	0.10
SPM10	3	0.10	0.08	0.07	SPM11	3	0.15	0.12	0.07	SPM12	3	0.23	0.03	0.06
SPM10	4	0.13	0.00	0.06	SPM11	4	0.14	0.00	0.08	SPM12	4	0.12	0.00	0.07
SPM10	5	0.10	0.08	0.06	SPM11	5	0.08	0.19	0.07	SPM12	5	0.14	0.00	0.06
SPM10	6	0.10	0.32	0.03	SPM11	6	0.07	0.00	0.07	SPM12	6	0.07	0.20	0.07
SPM10	7	0.09	0.00	0.00	SPM11	7	0.06	0.00	0.01	SPM12	7	0.08	0.00	0.02

*Note:* Cat = category.

## Abbreviations

The following abbreviations are used in this manuscript:

AIC	Akaike information criterion
BIC	Bayesian information criterion
EM	expectation maximization
LASSO	least absolute shrinkage and selection operator
LCA	latent class analysis
LCM	latent class model
NLM	nested logit model
RLCA	regularized latent class analysis
RLCM	restricted latent class model
SPM-LS	last series of Raven’s standard progressive matrices

## Appendix A. Additional Results for Simulated Data Illustration with Polytomous Item Responses

In Table A1, estimated item response probabilities for the exploratory LCM with four latent classes are shown.

**Table A1:** Data illustration polytomous data: estimated item response probabilities in the exploratory LCM with C=4 classes.

Item	Cat	Class				Item	Cat	Class				Item	Cat	Class			
		1	2	3	4			1	2	3	4			1	2	3	4
1	0	0.08	0.78	0.80	0.82	5	0	0.10	0.11	0.45	0.92	9	0	0.14	0.82	0.14	0.81
1	1	0.33	0.08	0.07	0.07	5	1	0.34	0.27	0.23	0.02	9	1	0.27	0.08	0.26	0.08
1	2	0.31	0.05	0.05	0.06	5	2	0.32	0.31	0.17	0.02	9	2	0.32	0.05	0.33	0.05
1	3	0.29	0.08	0.08	0.06	5	3	0.24	0.31	0.15	0.04	9	3	0.27	0.05	0.27	0.05
2	0	0.20	0.84	0.89	0.91	6	0	0.22	0.24	0.31	0.77	10	0	0.19	0.89	0.40	0.83
2	1	0.30	0.06	0.00	0.06	6	1	0.23	0.17	0.16	0.07	10	1	0.42	0.05	0.28	0.05
2	2	0.28	0.06	0.01	0.03	6	2	0.23	0.21	0.06	0.03	10	2	0.19	0.01	0.12	0.03
2	3	0.23	0.03	0.10	0.00	6	3	0.31	0.39	0.47	0.13	10	3	0.20	0.05	0.20	0.10
3	0	0.15	0.76	0.20	0.80	7	0	0.07	0.79	0.83	0.84	11	0	0.10	0.13	0.55	0.90
3	1	0.25	0.15	0.34	0.10	7	1	0.34	0.10	0.06	0.05	11	1	0.32	0.28	0.10	0.03
3	2	0.36	0.06	0.18	0.05	7	2	0.31	0.07	0.06	0.06	11	2	0.26	0.34	0.17	0.03
3	3	0.24	0.03	0.28	0.06	7	3	0.28	0.03	0.06	0.05	11	3	0.32	0.25	0.18	0.04
4	0	0.27	0.89	0.30	0.86	8	0	0.24	0.86	0.91	0.88	12	0	0.25	0.19	0.21	0.77
4	1	0.35	0.04	0.28	0.04	8	1	0.23	0.06	0.05	0.06	12	1	0.23	0.24	0.27	0.08
4	2	0.19	0.02	0.21	0.01	8	2	0.24	0.06	0.02	0.06	12	2	0.19	0.20	0.10	0.04
4	3	0.19	0.05	0.22	0.09	8	3	0.28	0.02	0.03	0.00	12	3	0.34	0.38	0.42	0.12

*Note:* Cat = category.

## Appendix B. Additional Results for SPM-LS Dataset with Polytomous Item Responses

Table A2 shows all estimated item response probabilities for the SPM-LS dataset with polytomous items for the best fitting RLCM with C=3 classes.

**Table A2:** SPM-LS polytomous data: estimated item response probabilities and latent class probabilities for best-fitting RLCM with C=3 latent classes.

Item	Cat	C1	C2	C3	Item	Cat	C1	C2	C3	Item	Cat	C1	C2	C3
SPM1	0	0.73	0.15	0.82	SPM5	0	0.72	0.04	0.98	SPM9	0	0.25	0.22	0.73
SPM1	1	0.11	0.48	0.12	SPM5	1	0.05	0.48	0.00	SPM9	1	0.14	0.03	0.12
SPM1	2	0.06	0.01	0.02	SPM5	2	0.03	0.32	0.01	SPM9	2	0.17	0.00	0.07
SPM1	3	0.07	0.04	0.01	SPM5	3	0.08	0.00	0.00	SPM9	3	0.12	0.32	0.03
SPM1	4	0.00	0.24	0.01	SPM5	4	0.06	0.00	0.00	SPM9	4	0.19	0.15	0.01
SPM1	5	0.01	0.08	0.02	SPM5	5	0.02	0.08	0.01	SPM9	5	0.06	0.00	0.03
SPM1	6	0.02	0.00	0.00	SPM5	6	0.02	0.08	0.00	SPM9	6	0.06	0.16	0.01
SPM1	7	0.00	0.00	0.00	SPM5	7	0.02	0.00	0.00	SPM9	7	0.01	0.12	0.00
SPM2	0	0.87	0.32	0.97	SPM6	0	0.51	0.08	0.92	SPM10	0	0.08	0.00	0.56
SPM2	1	0.02	0.12	0.03	SPM6	1	0.10	0.36	0.04	SPM10	1	0.14	0.12	0.19
SPM2	2	0.02	0.36	0.00	SPM6	2	0.11	0.00	0.03	SPM10	2	0.26	0.40	0.03
SPM2	3	0.07	0.00	0.00	SPM6	3	0.09	0.00	0.01	SPM10	3	0.10	0.08	0.07
SPM2	4	0.01	0.12	0.00	SPM6	4	0.06	0.24	0.00	SPM10	4	0.13	0.00	0.06
SPM2	5	0.00	0.08	0.00	SPM6	5	0.06	0.20	0.00	SPM10	5	0.10	0.08	0.06
SPM2	6	0.01	0.00	0.00	SPM6	6	0.05	0.08	0.00	SPM10	6	0.10	0.32	0.03
SPM2	7	0.00	0.00	0.00	SPM6	7	0.02	0.04	0.00	SPM10	7	0.09	0.00	0.00
SPM3	0	0.67	0.04	0.92	SPM7	0	0.38	0.20	0.88	SPM11	0	0.11	0.26	0.48
SPM3	1	0.17	0.00	0.05	SPM7	1	0.06	0.12	0.06	SPM11	1	0.18	0.43	0.10
SPM3	2	0.08	0.40	0.00	SPM7	2	0.13	0.28	0.01	SPM11	2	0.21	0.00	0.12
SPM3	3	0.01	0.32	0.00	SPM7	3	0.12	0.28	0.01	SPM11	3	0.15	0.12	0.07
SPM3	4	0.01	0.04	0.02	SPM7	4	0.11	0.00	0.02	SPM11	4	0.14	0.00	0.08
SPM3	5	0.00	0.12	0.01	SPM7	5	0.07	0.00	0.02	SPM11	5	0.08	0.19	0.07
SPM3	6	0.04	0.04	0.00	SPM7	6	0.09	0.00	0.00	SPM11	6	0.07	0.00	0.07
SPM3	7	0.02	0.04	0.00	SPM7	7	0.04	0.12	0.00	SPM11	7	0.06	0.00	0.01
SPM4	0	0.62	0.00	0.98	SPM8	0	0.18	0.00	0.81	SPM12	0	0.03	0.24	0.45
SPM4	1	0.11	0.40	0.01	SPM8	1	0.24	0.04	0.01	SPM12	1	0.14	0.09	0.17
SPM4	2	0.04	0.36	0.00	SPM8	2	0.08	0.00	0.07	SPM12	2	0.19	0.44	0.10
SPM4	3	0.07	0.08	0.00	SPM8	3	0.14	0.08	0.03	SPM12	3	0.23	0.03	0.06
SPM4	4	0.05	0.00	0.01	SPM8	4	0.11	0.20	0.03	SPM12	4	0.12	0.00	0.07
SPM4	5	0.04	0.12	0.00	SPM8	5	0.07	0.28	0.04	SPM12	5	0.14	0.00	0.06
SPM4	6	0.04	0.00	0.00	SPM8	6	0.11	0.40	0.01	SPM12	6	0.07	0.20	0.07
SPM4	7	0.03	0.04	0.00	SPM8	7	0.07	0.00	0.00	SPM12	7	0.08	0.00	0.02

*Note:* Cat = category.
